# MicroRNA *MIMIC* binding sites: Minor flanking nucleotide alterations can strongly impact *MIMIC* silencing efficacy in Arabidopsis

**DOI:** 10.1002/pld3.88

**Published:** 2018-10-23

**Authors:** Gigi Wong, Maria Alonso‐Peral, Bingjun Li, Junyan Li, Anthony A. Millar

**Affiliations:** ^1^ Division of Plant Science Research School of Biology The Australian National University Canberra Australian Capital Territory Australia

**Keywords:** Arabidopsis, *MIMIC*s, miR159, miR165/166, miRNAs

## Abstract

In plants, microRNA (miRNA) target *MIMIC*s (*MIM*s) have been widely used to inhibit miRNA function. They are based on the Arabidopsis *INSENSITIVE TO PHOSPATE STARVATION 1* (*IPS1*) gene that corresponds to a non‐coding RNA containing a miR399 binding site that can be modified to sequester and inhibit any miRNA of interest. However, the efficacy of miRNA inhibition of these different *MIM*s can vary greatly. Using *MIM*s that have strong efficacy (*MIM159*) and poor efficacy (*MIM165*), we investigate the underlying cause of this variation. Firstly, sequence alignments of *IPS1* homologs from the *Brassicaceae* identified a highly conserved sequence immediately downstream of the miRNA binding site. Mutating this sequence in the context of the *MIM159* attenuates its strong efficacy. This conserved flanking region contains a predicted stem‐loop structure that is also predicted to be present in most modified *MIM*s that appear to have a strong efficacy, but not in *MIM165* that has a poor efficacy. Restoring this predicted stem‐loop in *MIM165* via mutation of only three or five nucleotides within the conserved flanking region resulted in *MIM165* variants that have very strong efficacies of miRNA inhibition. However, specifically mutating this predicted stem‐loop in the *MIM159* context failed to significantly reduce efficacy, and additional mutations to restore this predicted stem‐loop weakened efficacy further. Although this shows there is no simple correlation between this predicted stem‐loop and efficacy, these results add to the growing evidence that the sequence context of miRNA binding sites is important, and that minor nucleotide substitutions to flanking sequences of miRNA binding sites can strongly enhance or attenuate the miRNA‐target interaction.

## 1 INTRODUCTION

Plant microRNAs (miRNAs) are a class of small RNAs (20–24 nts) that are key negative regulators of gene expression, controlling diverse traits such as development, response to environmental stimuli, and plant‐microbe interactions (Li, Reichel, Li, & Millar, 2014). They operate by guiding the RNA induced silencing complex (RISC) to target mRNAs of high complementarity, where they repress expression through mRNA degradation (mRNA cleavage) or via a translational repression mechanism (Iwakawa & Tomari, 2015). Unlike animal systems, where miRNA‐target pairs can tolerate many mis‐matches, most canonical plant miRNA‐target pairs only contain a small number of mis‐matches, implying high complementarity is a strict requirement for repression in plants (Liu, Wang, & Axtell, 2014; Schwab et al., 2005). This constraint of high complementarity has remained a corner stone of plant miRNA biology, being the driving parameter of numerous bioinformatic approaches related to plant miRNAs (Dai, Zhuang, & Zhao, 2018).

In animal systems, it is clear that factors in addition to complementarity, such as RNA secondary structure or RNA‐binding proteins, can strongly impact the silencing outcome of miRNA‐target pair interactions (Kertesz, Iovino, Unnerstall, Gaul, & Segal, 2007; van Kouwenhove, Kedde, & Agami, 2011). By contrast, such evidence in plants is limited. However, we recently demonstrated that RNA secondary structure plays a role in controlling the regulation of the Arabidopsis *MYB33* gene, a conserved target of miR159. In this study, the flanking sequences of the miR159‐binding site of *MYB33* were shown to form conserved RNA secondary structures that were required for strong silencing (Li, Reichel, & Millar, 2014; Zheng et al., 2017). This demonstrates that sequence context of a miRNA binding site can strongly impact the silencing outcome, possibly through favorable RNA secondary structure that promotes miRNA‐target recognition.

We have previously argued that such factors beyond complementarity may underlie a much narrower functional specificity of plant miRNAs than that of bioinformatically predicted targets that are based primarily on complementarity (Li, Reichel, Li et al., 2014). Such a principle may also impact the efficacy of artificial miRNAs (amiRNA) or miRNA decoys, such as target *MIMIC*s (*MIM*s), *SPONGE*s (*SP*s), and *Short Tandem Target MIMIC*s (*STTM*s). For instance, different amiRNAs that have analogous complementarities to their target mRNAs work with considerable variability that cannot be explained by complementarity alone (Deveson, Li, & Millar, 2013; Li et al., 2013). Similarly, identical miRNA binding sites embedded within different decoy backbones, inhibit miRNA function with highly variable efficacies (Reichel, Li, Li, & Millar, 2015; Yan et al., 2012). In appraising the efficacies of *MIM*s, *SP*s, and *STTM*s to inhibit two different miRNA families, miR159 and miR165, the efficacy of the approaches was highly variable (Reichel et al., 2015). For example, *MIM159* could inhibit miR159 much more strongly than either a *STTM159* or *SP159* approach. Conversely, *SP165* and *STTM165* inhibited miR165 much more efficiently than *MIM165* (Reichel et al., 2015). Therefore, no one single decoy approach could guarantee strong miRNA inhibition. Not only does the same binding site within different backbones result in variable efficacies, but also different binding sites within the same backbone, such as the contrasting strong efficacy of *MIM159* to the weak efficacy of *MIM165* (Reichel et al., 2015). Given the current broad use of different miRNA decoy approaches to modulate and understand the miRNA function (Zhang et al., 2017; Zhao et al., 2016, 2017), understanding the underlying mechanism(s) responsible for decoy efficacy will be important for their optimal use. Here, we focus on the widely used *MIM*s, and find their efficacy of miRNA inhibition can be strongly impacted by subtle changes to flanking nucleotide composition.

## 2 MATERIALS AND METHODS

### 2.1 Plant material and growth conditions

All plant lines were in the *Arabidopsis thaliana* Columbia‐0 (Col‐0) background. Seeds were vapor sterilized using chlorine gas by mixing 100 ml of 100% sodium hypochlorite with 3 ml of 36% HCl. Seeds were sown on MS medium agar plates with hygromycin selection. At 7–10 days old, seedlings were transferred to soil (Debco plugger soil mix with 3.5 g/L Osmocote fertiliser). All seeds were stratified for 24 hr at 4°C in the dark prior to growth in “long day” conditions (16 hr light/8 hr dark cycle) under fluorescent illumination of 150–200 μmol/m^2^/s at 22°C.

### 2.2 Generation of *MIM* constructs and transformation into Arabidopsis

All *MIM159* and *MIM165* constructs were synthesized (Integrated DNA Technologies) with Gateway attB1 and attB2 sites (Invitrogen) harboring *MIM* binding sites as designed by Todesco, Rubio‐Somoza, Paz‐Ares, and Weigel (2010). Sequences were cloned into the Gateway entry vector, pDONR/Zeo (Invitrogen), using Gateway BP Clonase II (Invitrogen) and verified using restriction enzyme digestion analysis and sequencing. Resulting plasmids were then recombined into the Gateway compatible binary vector, pMDC32 (Curtis & Grossniklaus, 2003) using Gateway LR Clonase II (Invitrogen). Binary vectors were verified using restriction enzyme digestion analysis and were then transformed into *Agrobacteria tumefaciens* strain GV3101 (Hellens, Mullineaux, & Klee, 2000) by electroporation. Plasmids were extracted for verification using restriction enzyme digestion analysis. *MIM159* and *MIM165* constructs were transformed into Col‐0 using the floral dipping method (Clough & Bent, 1998).

### 2.3 qRT‐PCR

Total RNA was extracted from 4‐week‐old rosettes using Trizol reagent (Invitrogen). For all *MIM159* constructs, 14 rosettes from independent lines were pooled together for RNA extraction to represent one biological replicate. Three biological replicates were used per construct. Twenty microgram of total RNA was RQ1 DNase treated (Promega) with the addition of 40 units/20 μl RNaseOUT Recombinant RNase inhibitor (Invitrogen). DNase treated RNA was purified using RNeasy Mini kit (QIAGEN) and RNA quality analyzed on 1% agarose gel. 500 ng–2 μg of treated RNA was used for reverse transcription with Superscript III Reverse Transcriptase (Invitrogen) using the oligo dT primer (Invitrogen). cDNA was diluted to 50‐fold in nuclease‐free water and used for qRT‐PCR. *CYCLOPHILIN 5* (At2 g29960) was also measured to normalize the total amount of cDNA input allowing for comparative quantitation using the Rotor‐Gene Q software package. The following gene specific primers were used to amplify *MIM159* and *MIM165* constructs for qRT‐PCR [5′‐CATTATGTTTGGGTTGTACC][5′‐GCACTGGTCTGACTATTCTCC]. *OsMIM159* was amplified using the following primers [5′‐TCTCAAAGAGGCACCAATAC][5′‐ATAATGTGGAGTGTGCCCTG]. qRT‐PCR was performed on a Rotor‐Gene Q real‐time PCR machine (QIAGEN) using three technical replicates. The following cycle conditions were used: 1 cycle of 95°C/5 min, 45 cycles of 95°C/15 s, 60°C/15 s, and 72°C/20 s of fluorescence was acquired at the 72°C step. A 55–99°C melting cycle was then performed.

### 2.4 Bioinformatic alignments and prediction of RNA secondary structure

Gene sequences were obtained using the Basic Local Alignment Search Tool (BLAST), and aligned using the alignment software MAFFT (Katoh, Misawa, Kuma, & Miyata, 2002). Bioinformatic prediction of RNA secondary structure of *MIMs* was calculated using the Vienna RNAfold WebServer, (http://rna.tbi.univie.ac.at/cgi-bin/RNAWebSuite/RNAfold.cgi; Hofacker, 2003). All RNAfold analyses were performed using the Turner model, 2004 energy parameter (Mathews et al., 2004) at 22°C. Whole sequences were used for all RNA secondary structure prediction.

### 2.5 Statistical analysis

Plant morphological phenotyping was analyzed using Pearson Chi‐Squared tests. qRT‐PCR data for *MIM159* were analyzed using analysis of variance (ANOVA) on log transformed relative expression followed by a post hoc multiple comparisons using Tukey's HSD. Data were log transformed to homogenize the standard deviations.

## 3 RESULTS

### 3.1 Nucleotides flanking the miR399 binding sites of *IPS1* homologs are highly conserved

Target *MIM*s are based on the non‐coding RNA, *INSENSITIVE TO PHOSPHATE STARVATION 1* (*IPS1*) from Arabidopsis. It contains a miR399 binding site with a central 3 nt bulge to prevent slicing, where it is thought that miR399 irreversibly binds *IPS1* resulting in miR399 sequestration and functional inhibition (Franco‐Zorrilla et al., 2007). Replacing the *IPS1* miR399 binding site with sequences that target other miRNAs is able to inhibit a wide range of different miRNAs, making this *MIM* approach a versatile functional tool (Franco‐Zorrilla et al., 2007; Todesco et al., 2010).

**Figure 1:**
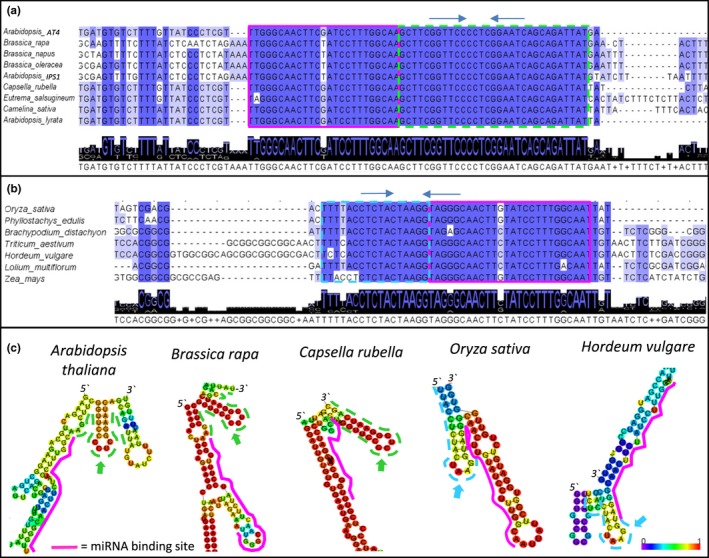
*IPS1* homologs have conserved nucleotides flanking the binding site that contain putative stem‐loop regions. *IPS1* homologs were aligned using MAFFT. Conserved nucleotide sequences are observed in (a) *Brassicaceae* family members (green dashed box) and (b) various monocotyledonous plants (light blue dashed box). The binding site is indicated in the red box. Putative RNA stems in conserved sequences are indicated with arrows. (c) Predicted RNA secondary structures of *IPS1* homologs from the *Brassicaceae* family and monocotyledonous plants as determined using the RNAfold WebServer. The miR399 binding site is indicated by the pink line and the conserved sequence in green dashed lines for the *Brassicaceae IPS1* homologs and blue dashed lines for the monocotyledonous homologs. Arrows of the respective colors indicate the predicted stem‐loop. Color of nucleotides represents the probability of structure formation

*IPS1* homologs are present in diverse monocot and dicot species of plants, and although *IPS1* homologs are non‐coding RNAs, their length is consistently over 500 nt (Table S1), suggesting the maintenance of such a lengthy backbone signifies functional importance. However, other than the 23‐nt miR399 binding site, very little sequence conservation is shared amongst *IPS1* homologs from these diverse species of plants (Franco‐Zorrilla et al., 2007; Huang, Shirley, Genc, Shi, & Langridge, 2011; Supporting Information Figure S1). By contrast, when alignments were performed with *IPS1* homologs from just the *Brassicaceae* family, three strongly conserved regions were identified (Supporting Information Figure S2). Most notably was a fully conserved 32‐nt region immediately downstream of the miR399 binding site in the *Brassicaceae IPS1* homologs (Figure 1a). Similarly, an alignment of monocot *IPS1* homologs identified a 12‐nt region immediately upstream of the miR399 binding site that was strongly conserved (Figure 1b), when compared to the other regions of *IPS1* homologs from monocot species (Supporting Information Figure S3). The proximity of these conserved sequences to the miR399 binding site suggests they may be playing a role in the *IPS1*–miR399 interaction.

### 3.2 The *IPS1* backbone sequence impacts the efficacy of miRNA inhibition

**Figure 2:**
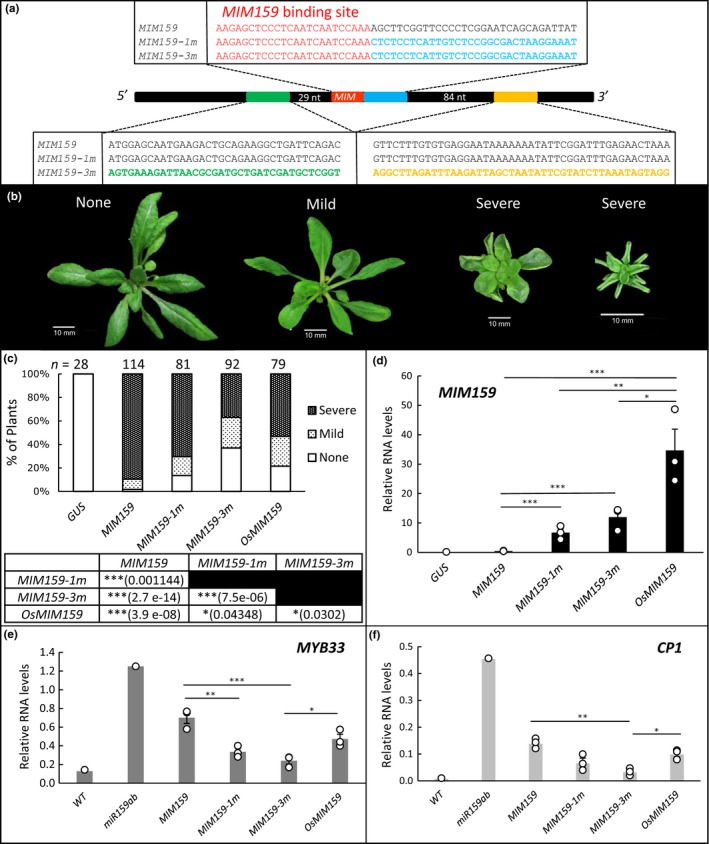
Generation and analysis of different *MIM159* transgenes. (a) *MIM159* was mutated in the downstream conserved flanking region of the miRNA binding site (in red) to create *MIM159‐1m* (in blue). Two further mutations were made in the conserved regions further downstream and upstream of the miR159 binding site (in orange and green, respectively) to create *MIM159‐3m*. (b) Rosette phenotypes were scored from 4‐wk‐old transformed plants. Phenotypic categories of “None” (phenotype indistinguishable from WT), “Mild” (presence of leaf curvature), and “Severe” (Leaves strongly curled so that the abaxial side is showing of two or more leaves) plant phenotypes. (c) Percentage of plants displaying each category of phenotypes as shown in B. Significance values are shown, calculated using Pearson Chi‐Squared tests. (d‐f) Transcript profiling of (d) the different *MIMs*, (E) *MYB33*, and (e) *CP1*. Measurements are composed of three biological replicates (shown as dots), with each replicate being composed of 14 randomly selected primary transformants. Statistical analysis was performed using analysis of variance where significant differences between means are indicated by ‘*’ *p* < 0.05, ‘**’ *p* < 0.01 and ‘***’ *p* < 0.001. Error bars represent the standard error of the mean

To test whether these three conserved regions impact the ability of Arabidopsis *IPS1* to inhibit miRNA function, we utilized *MIM159*, a modified *IPS1* in which the binding site has been changed to target miR159 (Todesco et al., 2010). Expression of *MIM159* in Arabidopsis results in characteristic phenotypic defects of the rosette whose severity can be readily assessed to gauge the extent of miR159 inhibition (Reichel et al., 2015). We mutated the nucleotide region immediately adjacent to the miRNA binding site of *MIM159* to a different sequence (but maintaining overall GC content) to result in the variant, *MIM159‐1m* (Figure 2a). Additional mutations were then made to the other two conserved regions to generate the variant *MIM159‐3m*, so that it contained three different mutated regions (Figure 2a). Finally, a *MIM159* binding site was incorporated into *IPS1* from *Oryza sativa,* designated as *OsMIM159*. *OsIPS1* has little sequence identity to *Arabidopsis IPS1*, where the miR399 binding site is the only strongly conserved region between these two *IPS1* homologs (Supporting Information Figure S4). The performance of these three *MIM*s were then compared against the Arabidopsis *MIM159* transgene.

All *MIM*s were cloned into the vector pMDC32, so to be under the transcriptional control of the 2×35S promoter (Curtis & Grossniklaus, 2003). After transformation into Arabidopsis, the rosette phenotype of multiple independent transformants was scored. Phenotypes were classified as either No Phenotypic (N; indistinguishable from wild‐type), Mild (M; presence of leaf curvature), and Severe (S; the plant has strongly curled leaves with the abaxial side of two or more leaves being visible from an aerial view) (Figure 2b). The relative proportions of these phenotypic categories were used as a measure of silencing efficacy of the different *MIM* transgenes. A *35S:GUS* construct was used as a transgenic negative control.

As previously reported, *MIM159* displays a strong efficacy, as the majority of plants [102/114 (89%)] display “severe” phenotypic defects (Figure 2c). By contrast, *MIM159‐1m* transformants display “severe” phenotypes at a significantly lower frequency [57/81 (70%), *p* = 0.001144]. Furthermore, *MIM159‐3m* transformants displayed even fewer plants with “severe” phenotypes [34/92 (37%)], being significantly different from *MIM159‐1m* (*p* = 7.476e‐06). Consistent with our previous data (Reichel et al., 2015), this demonstrates that sequences outside of this miRNA binding site impacts the silencing efficacy of the *MIM159* construct. Furthermore, the frequency of *OsMIM159* transformants that displayed severe phenotypes was significantly lower than *MIM159* [42/79 (53%), *p *= 3.988e‐08] (Figure 2c). Therefore, changing the *IPS1* backbone changes the silencing efficacy of the *MIM*.

To further analyze these plants, we used qRT‐PCR to measure the transcript levels of a major target gene of miR159, *MYB33*, as well as a *MYB33* downstream gene, *CP1* (Alonso‐Peral et al., 2010). RNA was prepared from multiple randomly selected transformants and qRT‐PCR was performed. Generally, both the *MYB33* and *CP1* mRNA levels correlated with the strength of the phenotypic defects, with *MIM159* plants having the highest *MYB33*/*CP1* mRNA levels and *MIM159‐3m* having the lowest (Figure 2e–f). *OsMIM159* plants that had intermediate efficacy compared to *MIM159* and *MIM159‐3m*, contained intermediate levels of *MYB33* and *CP1* transcript levels (Figure 2e–f).

### 3.3 The mRNA levels of the *MIM*s inversely correlate with silencing efficacy

Although all the *MIM*s in the above experiment are all under the control of an identical promoter (2× 35S), it is possible that the different *MIM* transcripts have different RNA stabilities (steady‐state levels) which may explain their different efficacies. To investigate this, the RNA levels of the different *MIM*s were measured using qRT‐PCR. The primers used were identical for *MIM159*,* MIM159‐1m,* and *MIM159‐3m*, but differed for *OsMIM159*. As a transgenic negative control, *35S:GUS* transformants were used.

Strikingly, *MIM* transcript levels were generally inversely correlated to their silencing efficacies. *MIM159* had the lowest RNA level despite having the greatest efficacy, with *MIM159‐3m* plants having at least a tenfold higher levels of the *MIM* RNA (Figure 2d). Interestingly, *OsMIM159* plants had the highest *MIM* RNA level, despite this transgene having a weaker efficacy than *MIM159* (Figure 2c). These results argue against lower RNA levels being the cause of the reduced efficacy of the mutated *MIM159* transgenes or the relatively lower efficacy of *OsMIM159,* but rather other factors are in play. In fact, the inverse correlation may argue that if a *MIM* has a strong interaction with its target miRNA, this may promote *MIM* RNA degradation, so a low RNA level maybe reflective of strong miRNA‐*MIM* target recognition. Alternatively, the inverse correlation of *MIM* RNA levels to efficacy may suggest that their expression results in lethality and that only plants expressing *MIM*s below a certain level can be generated, where the greater the efficacy, the lower the lethal dose of *MIM* RNA.

### 3.4 Predicted RNA stem‐loop structures are adjacent to the miR399 binding site of *IPS1*

Recently, we have shown that a RNA stem‐loop structure that is adjacent to the miR159 binding site of *MYB33* is required for strong miR159‐mediated silencing (Zheng et al., 2017). Interestingly, within the conserved 32‐nt region adjacent to the miR399 binding site of *IPS1 Brassicaceae* homologs, a stem‐loop structure of high confidence is predicted to form as determined by the Vienna RNAfold WebServer (Figure 1c). Similarly, within the conserved stretch of nucleotides adjacent to the miR399 binding site of *IPS1* genes from monocotyledonous plants, a stem‐loop structure is also predicted to form, albeit with less confidence (Figure 1c).

**Figure 3:**
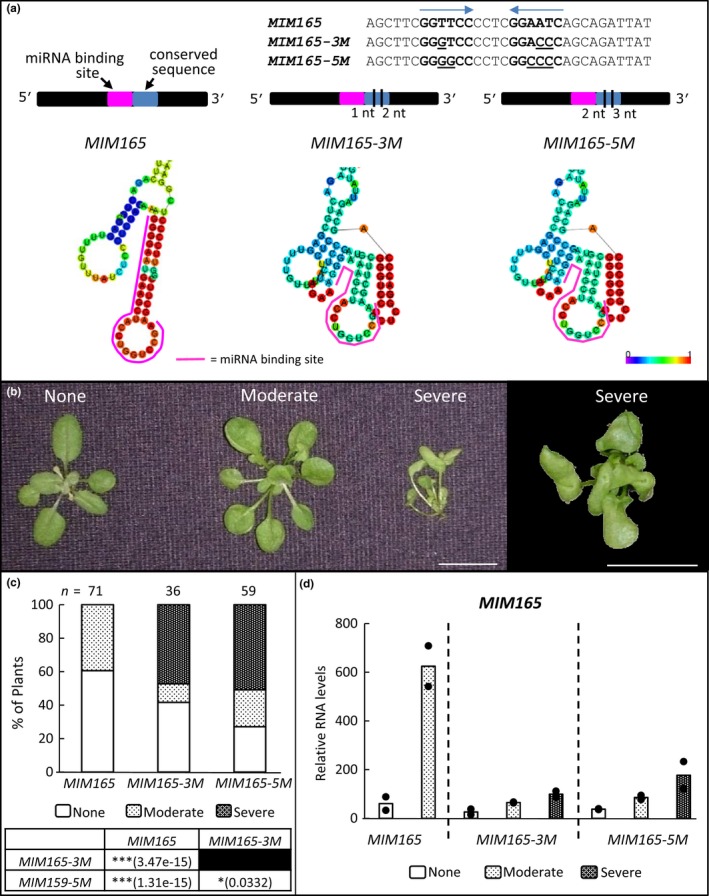
Generation and analysis of the *MIM165*,*MIM165‐3M* and *MIM165‐5M* transgenes. (a) Sequence of the conserved flanking region of original *MIM165* and the two variants, *MIM165‐3M* and *MIM165‐5M*, with the putative stem‐loop in bold nucleotides (also indicated with arrow) and mutations underlined. Black lines in the cartoons of the transgenes also indicate the position and number of mutations made. The predicted secondary structure of *MIM165* and *MIM165* mutants is shown, with the miR165 binding site indicated by a pink line. (b) Phenotypic categories of “None” (indistinguishable from wild‐type), “Mild” (evidence of adaxialized leaves), and “Severe” (trumpet or cup‐shaped leaves) plant phenotypes. (c) Percentage of plants displaying each category of phenotypes from the three *MIM165* constructs. Significance values are shown, calculated using Pearson Chi‐Squared tests. (d) Relative *MIM165* RNA levels in transgenic plants from different phenotypic categories of the different *MIM165* variants. Measurements are from two biological replicates (shown as dots), each being composed of five primary transformant plants

Similar to the case for *MYB33*, we investigated whether this potential RNA stem‐loop promoted miRNA‐target interaction. We have previously shown that *MIM*s harboring different miRNA binding sites display dramatically different efficacies in inhibiting their target miRNAs (Reichel et al., 2015). Given that changing the miRNA binding site within a given context can alter local RNA secondary structures, we determined the predicted RNA secondary structure for all the *MIM*s whose expression could result in phenotypic defects (Supporting Information Figure S5; Todesco et al., 2010). We found that most *MIM*s contained the identical predicted RNA stem‐loop arising from the conserved adjacent region of *IPS1*, although with varying degrees of confidence. However, *MIM165* was not predicted to contain this stem‐loop structure (Figure 3a), but instead the stem‐loop nucleotides are predicted to base‐pair with the *MIM165* binding site, possibly attenuating the binding site's accessibility. As *MIM165* has been previously reported to have poor silencing efficacy (Reichel et al., 2015; Yan et al., 2012), we investigated whether the efficacy of *MIM165* could be increased via restoring the predicted RNA stem‐loop structure.

### 3.5 Restoring the predicted RNA stem‐loop structure in *MIM165* transforms its silencing efficacy

Based on RNA secondary structure prediction using the Vienna RNAfold WebServer, we generated two different *MIM165* variants, designated *MIM165‐3M* and *MIM165‐5M*, that had three and five nt alterations in the flanking region of the *MIM165* binding site that was predicted to restore the stem‐loop structure and also change the secondary structure of the *MIM165* binding site itself (Figure 3a). The idea was to introduce mutations that would strengthen the stem‐loop through additional G‐C pairs, with *MIM165‐5M* having a G‐C pair replace an A‐U pair in the stem (Figure 3a). The stem‐loop was predicted to be present when the entire *MIM165* RNAs were folded (Supporting Information Figure S6). The *MIM165* binding sites of the two variants remained unchanged from the binding site of the parental *MIM165* transgene, and all three binding sites were identical to the STTM165 binding site (Yan et al., 2012), including the three nucleotide bulge (CTA) at position 10–11 (Todesco et al., 2010). The three different *MIM165* constructs were independently transformed into wild‐type plants and their efficacies scored based on the frequency and severity of phenotypes of primary transformants. Plants were either classified as having phenotypes with; None (indistinguishable from wild‐type), Mild (evidence of adaxialized leaves), or severe (trumpet or cup‐shaped leaves) defects (Figure 3b). As has been previously found (Todesco et al., 2010; Yan et al., 2012), *MIM165* failed to result in any transgenic plants displaying a severe phenotype of trumpet or cup‐shaped leaves (Figure 3c). By contrast, many *MIM165‐3M* and *MIM165‐5M* transformants displayed severe phenotypes, with *MIM165‐5M* having the greatest proportion of plants with phenotypic defects. Therefore, seemingly minor nucleotide changes to the flanking regions of the *MIM165* binding site have had a strong impact on the efficacy of this *MIM*.

In order to gain insight into the mechanism behind this increased efficacy, we measured *MIM165* RNA levels in the different phenotypic categories for each of the three *MIM165* transgenes. For *MIM165‐3M* and *MIM165‐5M*, the *MIM165* RNA levels followed a simple trend of directly correlating with phenotypic severity, where the stronger the phenotype, the higher the *MIM165* transcript level (Figure 3d). However, for the parental *MIM165* transgene, much higher *MIM165* RNA levels were found in plants with Mild defects in comparison to *MIM165‐3M* and *MIM165‐5M* plants that displayed similar Mild defects (Figure 3d). As these steady‐state RNA levels will depend on both transcription and degradation, we hypothesize that only in *MIM165* plants where the transgene is very strongly transcribed can phenotypic defects arise. Additionally, consistent with the different *MIM159* transgenes (Figure 2), *MIM*s that perform poorly have higher transcript levels, supporting the notion that if *MIM*–miRNA interactions are strong, this will promote *MIM* RNA degradation, or that overexpression of *MIM*s with strong efficacy lead to plant death.

### 3.6 The predicted RNA stem‐loop in *MIM159* does not correlate with strong efficacy

**Figure 4:**
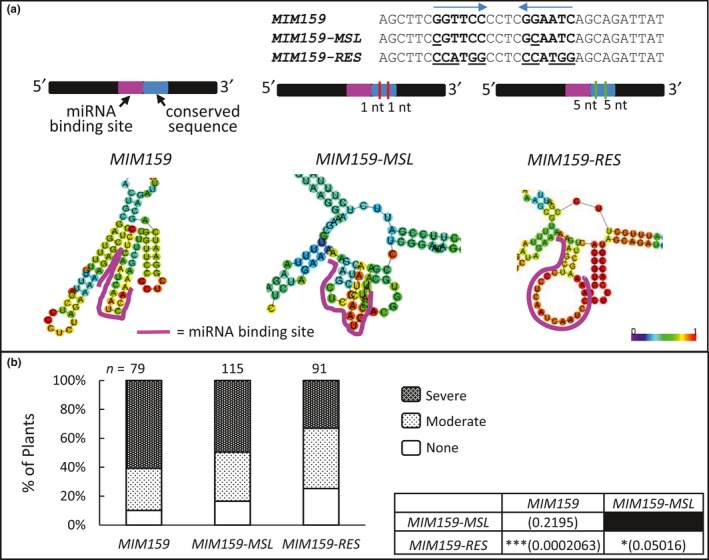
Generation and analysis of the *MIM159*,*MIM159‐MSL* and *MIM159‐RES* transgenes. (a) Sequence of the conserved flanking region of original *MIM159* and the two variants, *MIM159‐MSL* and *MIM159‐RES*, with the putative stem‐loop in bold nucleotides (also indicated with arrow) and mutations underlined. The cartoon depicts the original *MIM159* and the two variants *MIM159‐MSL* and *MIM159‐RES*. The red lines indicate the position and number of mutations made to destroy the stem‐loop in *MIM159‐MSL* and the green line for mutations made to restore the stem‐loop in *MIM159‐RES*. The predicted secondary structure of *MIM159* and *MIM159* mutants. The miR159 binding site is indicated by the pink line (b) Percentage of plants displaying each category of phenotypes from *MIM159* and *MIM159* mutants. The same phenotypic categories were used as for Figure 2b. Significance values are shown, calculated using Pearson Chi‐Squared tests

As the *MIM159‐1m* mutant no longer contains the predicted RNA stem‐loop, we investigated whether this predicted stem‐loop correlated with stronger efficacy of *MIM159*. Firstly, two nucleotide mutations were generated in *MIM159* to generate the construct *MIM159‐MSL*, which resulted in a construct predicted not to contain the stem‐loop (Figure 4a). However, although less *MIM159‐MSL* transformants displayed severe phenotypes compared to *MIM159* transformants, this difference was not statistically different (Figure 4b, [*p* = 0.2195]). Then, a second construct was generated, *MIM159‐RES*, in which further mutations were introduced that were predicted to restore the stem‐loop, but not the sequence (Figure 4a). However, *MIM159‐RES* displayed a weaker efficacy than *MIM159*, as there was significantly less *MIM159‐RES* transformants displaying severe phenotypes (Figure 4B, [*p* = 0.0002063]). This was despite the *MIM159* binding site in *MIM159‐RES* having a predicted RNA secondary structure that somewhat resembled the *MIM165‐3M* and *MIM165‐5M* transgenes (Figure 4a). Therefore, the presence or absence of this predicted stem‐loop is not an absolute indicator of the silencing performance of any given *MIM* decoy.

## 4 DISCUSSION

Previously, we assessed the capacity of *MIM*s, *STTM*s, and *SP*s regarding their ability to inhibit miRNA activity. Firstly, we found that no one approach guaranteed strong miRNA inhibition, where changing the binding site within the backbone altered their efficacies of miRNA inhibition. Secondly, that identical binding sites within different backbones worked with highly variable efficacies (Reichel et al., 2015). Both of these observations imply an interaction between the binding site and backbone sequences, which can either be favorable or detrimental to the decoy's silencing efficacy. However, the underlying reasons for this could be variable, including the steady‐state levels of the RNA decoy transcripts, the accessibility of the miRNA binding site within the decoy or other unknown mechanisms.

Here, we show subtle nucleotide changes adjacent to the miRNA binding site of *MIM*s can dramatically alter their efficacy of inhibition of miRNA function. This was most apparent for *MIM165*, where a three nucleotide change could transform its efficacy from a poor to highly effective miRNA decoy. Interestingly, the design of the alteration was to restore a small predicted stem‐loop that is present in a stretch of nucleotides that are conserved immediately adjacent to the miRNA binding site within *IPS1* homologs from the *Brassicaceae*.

From RNA secondary structure predictions, the restoration of this stem‐loop in *MIM165‐3M* and *MIM165‐5M* is predicted to prevent a strong RNA secondary structure that forms in the parental *MIM165* decoy that potentially sequesters much of the *MIM165* binding site (Figure 3a). Such alterations to RNA secondary structure are the most likely explanation underpinning the alteration of efficacy. Here, the mechanism may be, that if sequences flanking a miRNA binding site are in a secondary structure, they are less likely to base‐pair with the binding site itself, and hence increase accessibility of that binding site. Such a principle was hypothesized to be behind the strong efficacy of *STTM165/166* (Yan et al., 2012), where this decoy is composed of a 48 nt spacer region, that potentially forms a strong RNA secondary structure, that is flanked by two *MIM165* binding sites. Currently, the analysis of target site accessibility can only be derived from *in silico* prediction programs, where the standard approach is to analyze a region that includes 17 nt upstream and 13 downstream nucleotides of the miRNA binding site (Dai et al., 2018), a region that was found to correlate best with accessibility for animal miRNA‐target sites (Kertesz et al., 2007). However, the mutations that we have introduced into the *MIM*s here are outside of this region, as were the flanking mutations that impacted *MYB33* silencing (Zheng et al., 2017). Therefore, the flanking nucleotides included in such an analysis would need to be arbitrarily increased. Given all this uncertainty, it is unlikely that these *in silico* analyses are likely to generate informative predictions.

It is tempting to speculate that the presence of such a stem‐loop ensures strong efficacy of the *MIM* decoy, as most reported *MIM* constructs that result in miRNA inhibition have such a structure (Supporting Information Figure S5), as does *MIM165‐3M* and *MIM165‐5M* (Figure 3). However, mutation of this stem‐loop structure within the *MIM159* context, does not significantly attenuate efficacy, and additional compensatory mutations to restore the structure resulted in attenuated efficacy (Figure 4). Given the conflicting results between *MIM165* and *MIM159*, the presence or absence of the predicted stem‐loop cannot be regarded as an absolute indicator of efficacy. As mentioned above, the restoration of the stem‐loop in *MIM165‐3M* and *‐5M* may have abolished a competing RNA secondary structure that was predicted to form in *MIM165* (Figure 3a)*,* and which potentially inhibits the accessibility of the miRNA binding site. Therefore, in the *MIM165* context, the stem‐loop becomes an important determinant of silencing efficacy. By contrast for *MIM159*, no strong RNA secondary structure was predicted to sequester the miR159 binding site, even when the stem‐loop is abolished (Figure 4a). Therefore, the stem‐loop is not an important determinant of silencing efficacy in the *MIM159* context. Such a claim will need to be tested with further experimentation. Nevertheless, our observations highlight the complexity of miRNA binding sites, where changing only the miRNA binding site within the *MIM* backbone may not only change the miRNA that it targets, but potentially also the local RNA secondary structure that impacts miRNA‐target site accessibility, which ultimately impacts efficacy of the decoy.

Another possibility was that the mutations were altering the abundance of the decoys by altering their RNA stability, hence the more abundant the *MIM* was, the higher its efficacy. However, in all instances, the opposite appeared to be the case, where steady‐state levels of the *MIM*s inversely correlated with efficacy. For example, *MIM165‐3M* and *MIM165‐5M* required much lower steady‐state *MIM* transcript levels to induce moderate phenotypes compared to *MIM165* (Figure 3), indicating that the *MIM*s with higher efficacy can produce stronger phenotypes with lower expression levels. This would argue that miRNA‐*MIM* interaction is much more favorable with *MIM165‐3M* and *MIM165‐5M*, than for *MIM165*, as much less *MIM* RNA is required for the same silencing outcome. However, it is clear that the RNA expression level of the *MIM* is important for efficacy, where high enough expression may be able to overcome a weak *MIM*‐miRNA interaction. Expression of STTMs via a potato virus‐X system was able to strongly inhibit miR159 and miR165 in tobacco, where the STTM159 appeared to work even stronger than STTM165 based on relative miR159 and miR165 levels (Zhao et al., 2016).

In addition to sequences adjacent to the miRNA binding site, altering sequences further away, as is the case for *MIM159‐3m*, also altered the performance of the decoy. How the alteration of these sequences impacts the *MIM* efficacy is harder to explain, but as these sequences were more conserved in *IPS1* homologs from the *Brassicaceae*, it could be that they play some important role for inhibition of the miRNA that is yet to be identified.

In summary, we found that subtle mutations in flanking sequences could result in dramatic impacts on the performance of these *MIM*s. We speculate this is most likely due to alterations of RNA secondary structure and associated changes to miRNA binding site accessibility. Although we have analyzed artificial *MIM*s in this study, there is no reason why this principle will not apply to endogenous target genes or endogenous targets *MIM*s, for which many have been predicted (Dai et al., 2018; : Meng, Shao, Wang, & Jin, 2012), but how many of these interactions are functionally relevant remains a pressing question (Li, Reichel, Li et al., 2014). Currently, *MYB33* is one example in which conserved flanking nucleotides facilitates physiologically relevant silencing (Zheng et al., 2017). How many more natural miRNA targets are subject to such phenomenon remain to be determined. Currently, it appears *in silico* predictions of RNA secondary structure/miRNA binding site accessibility are so limited, they are unable to accurately assist in identifying favorable miRNA‐target interactions. Until such issues are resolved, identifying favorable miRNA‐target interactions will remain elusive and their discovery will be through laborious experimentation.

## AUTHOR CONTRIBUTIONS

G.W designed and performed experiments in Figures 1, 2 and 4. M.A‐P. and B.L. designed and performed experiments in Figure 3. J.L. and A.A.M supervised the project.

## References

[pld388-bib-0001] Alonso‐Peral, M. M. , Li, J. , Li, Y. , Allen, R. S. , Schnippenkoetter, W. , Ohms, S. , … Millar, A. A. (2010). The microR159 regulated *GAMYB‐like* genes inhibit growth and promote programmed cell death in Arabidopsis. Plant Physiology, 154, 757–771. 10.1104/pp.110.160630 20699403 PMC2949021

[pld388-bib-0002] Clough, S. J. , & Bent, A. F. (1998). Floral dip: A simplified method for Agrobacterium‐mediated transformation of *Arabidopsis thaliana* . The Plant Journal, 16, 735–743. 10.1046/j.1365-313x.1998.00343.x 10069079

[pld388-bib-0003] Curtis, M. D. , & Grossniklaus, U. (2003). A gateway cloning vector set for high‐throughput functional analysis of genes in planta. Plant Physiology, 133, 462–469. 10.1104/pp.103.027979 14555774 PMC523872

[pld388-bib-0004] Dai, X. , Zhuang, Z. , & Zhao, P. X. (2018) psRNATarget: A plant small RNA target analysis server (2017 release). Nucleic Acids Research, 46: W49 to W54 10.1093/nar/gky316 29718424 PMC6030838

[pld388-bib-0005] Deveson, I. , Li, J. , & Millar, A. A. (2013). MicroRNAs with analogous target complementarities perform with highly variable efficacies in Arabidopsis. FEBS Letters, 587, 3703–3708. 10.1016/j.febslet.2013.09.037 24103298

[pld388-bib-0006] Franco‐Zorrilla, J. M. , Valli, A. , Todesco, M. , Mateos, I. , Puga, M. I. , Rubio‐Somoza, I. , … PazAres, J. (2007). Target mimicry provides a new mechanism for regulation of microRNA activity. Nature Genetics, 39, 1033–1037. 10.1038/ng2079 17643101

[pld388-bib-0007] Hellens, R. , Mullineaux, P. , & Klee, H. (2000). Technical Focus: A guide to Agrobacterium binary Ti vectors. Trends in Plant Science, 5, 446–451. 10.1016/S1360-1385(00)01740-4 11044722

[pld388-bib-0008] Hofacker, I. L. (2003). Vienna RNA secondary structure server. Nucleic Acids Research, 31, 3429–3431. 10.1093/nar/gkg599 12824340 PMC169005

[pld388-bib-0009] Huang, C. Y. , Shirley, N. , Genc, Y. , Shi, B. , & Langridge, P. (2011). Phosphate utilization efficiency correlates with expression of low‐affinity phosphate transporters and noncoding RNA, *IPS1*, in barley. Plant Physiology, 156, 1217–1229. 10.1104/pp.111.178459 21606317 PMC3135919

[pld388-bib-0010] Iwakawa, H. , & Tomari, Y. (2015). The functions of microRNAs: mRNA decay and translational repression. Trends in Cell Biology, 25, 651–665. 10.1016/j.tcb.2015.07.011 26437588

[pld388-bib-0011] Katoh, K. , Misawa, K. , Kuma, K. , & Miyata, T. (2002). MAFFT: A novel method for rapid multiple sequence alignment based on fast Fourier transform. Nucleic Acids Research, 30, 3059–3066. 10.1093/nar/gkf436 12136088 PMC135756

[pld388-bib-0012] Kertesz, M. , Iovino, N. , Unnerstall, U. , Gaul, U. , & Segal, E. (2007). The role of site accessibility in microRNA target recognition. Nature Genetics, 39, 1278–1284. 10.1038/ng2135 17893677

[pld388-bib-0013] van Kouwenhove, M. , Kedde, M. , & Agami, R. (2011). MicroRNA regulation by RNA‐binding proteins and its implications for cancer. Nature Reviews Cancer, 11, 644–656. 10.1038/nrc3107 21822212

[pld388-bib-0014] Li, J.‐F. , Chung, H. S. , Niu, Y. , Bush, J. , McCormack, M. , & Sheen, J. (2013). Comprehensive protein‐based artificial microRNA screens for effective gene silencing in plants. The Plant Cell, 25, 1507–1522. 10.1105/tpc.113.112235 23645631 PMC3694689

[pld388-bib-0015] Li, J. , Reichel, M. , Li, Y. , & Millar, A. A. (2014). The functional scope of plant microRNA‐mediated silencing. Trends in Plant Science, 19, 750–756. 10.1016/j.tplants.2014.08.006 25242049

[pld388-bib-0016] Li, J. , Reichel, M. , & Millar, A. A. (2014). Determinants beyond both complementarity and cleavage govern microR159 efficacy in Arabidopsis. PLoS Genetics, 10, e1004232 10.1371/journal.pgen.1004232 24626050 PMC3953016

[pld388-bib-0017] Liu, Q. , Wang, F. , & Axtell, M. J. (2014). Analysis of complementarity requirements for plant microRNA targeting using a *Nicotiana benthamiana* quantitative transient assay. The Plant Cell, 26, 741–753. 10.1105/tpc.113.120972 24510721 PMC3967037

[pld388-bib-0018] Mathews, D. H. , Disney, M. D. , Childs, J. L. , Schroeder, S. J. , Zuker, M. , & Turner, D. H. (2004). Incorporating chemical modification constraints into a dynamic programming algorithm for prediction of RNA secondary structure. Proceedings of the National Academy of Sciences, 101, 7287–7292. 10.1073/pnas.0401799101 PMC409911 15123812

[pld388-bib-0019] Meng, Y. , Shao, C. , Wang, H. , & Jin, Y. (2012). Target mimics: An embedded layer of microRNA‐involved gene regulatory networks in plants. BMC Genomics, 13, 197 10.1186/1471-2164-13-197 22613869 PMC3441763

[pld388-bib-0021] Reichel, M. , Li, Y. , Li, J. , & Millar, A. A. (2015). Inhibiting plant microRNA activity: Molecular *SPONGE*s, target *MIMIC*s and STTMs all display variable efficacies against target microRNAs. Plant Biotechnology Journal, 13, 915–926. 10.1111/pbi.12327 25600074

[pld388-bib-0022] Schwab, R. , Palatnik, J. F. , Riester, M. , Schommer, C. , Schmid, M. , & Weigel, D. (2005). Specific effects of microRNAs on the plant transcriptome. Developmental Cell, 8, 517–527. 10.1016/j.devcel.2005.01.018 15809034

[pld388-bib-0023] Todesco, M. , Rubio‐Somoza, I. , Paz‐Ares, J. , & Weigel, D. (2010). A collection of target mimics for comprehensive analysis of microRNA function in *Arabidopsis thaliana* . PLoS Genetics, 6, e1001031 10.1371/journal.pgen.1001031 20661442 PMC2908682

[pld388-bib-0024] Yan, J. , Gu, Y. , Jia, X. , Kang, W. , Pan, S. , Tang, X. , … Tang, G. (2012). Effective small RNA destruction by the expression of a short tandem target mimic in Arabidopsis. The Plant Cell, 24, 415–427. 10.1105/tpc.111.094144 22345490 PMC3315224

[pld388-bib-0025] Zhang, H. , Zhang, J. , Yan, J. , Gou, F. , Mao, Y. , Tang, G. , … Zhu, J. K. (2017). Short tandem target mimic rice lines uncover functions of miRNAs in regulating important agronomic traits. Proceedings of the National Academy of Sciences of the United States of America, 114, 5277–5282. 10.1073/pnas.1703752114 28461499 PMC5441788

[pld388-bib-0026] Zhao, J. , Liu, Q. , Hu, P. , Jia, Q. , Liu, N. , Yin, K. , … Liu, Y. (2016). An efficient potato virus X‐ based microRNA silencing system in *Nicotiana benthamiana* . Scientific Reports, 6, 20573 10.1038/srep20573 26837708 PMC4738334

[pld388-bib-0027] Zhao, Y. , Wen, H. , Teotia, S. , Du, Y. , Zhang, J. , Li, J. , … Zhao, Q. (2017). Suppression of microRNA159 impacts multiple agronomic traits in rice (*Oryza sativa* L.). BMC Plant Biology, 17, 215 10.1186/s12870-017-1171-7 29162059 PMC5699021

[pld388-bib-0028] Zheng, Z. , Reichel, M. , Deveson, I. , Wong, G. , Li, J. , & Millar, A. A. (2017). Target RNA secondary structure is a major determinant of miR159 efficacy. Plant Physiology, 174, 1764–1778. 10.1104/pp.16.01898 28515145 PMC5490886

