# Supplementary material for: MicroRNA MIMIC binding sites: Minor flanking nucleotide alterations can strongly impact MIMIC silencing efficacy in Arabidopsis
Source: Plant Direct. 2018 Oct 23;2(10):e00088. doi: 10.1002/pld3.88 (PMC6508833; doi:10.1002/pld3.88)

Table S1.

	Species	*IPS1* length (nt)
	Arabidopsis thaliana	542
Monocots	Oryza sativa	661
	Zea mays	623
	Triticum aestivum	729
	Hordeum vulgare	611*
Dicots	Vitis vinifera	572
	Populus trichocarpa	519
	Medicago tuncatulata	504
	Solanum lycopersicum	430
	Brassica napus	557
	Phaseolus vulgaris	536
	Glycine max	769

Figure S1

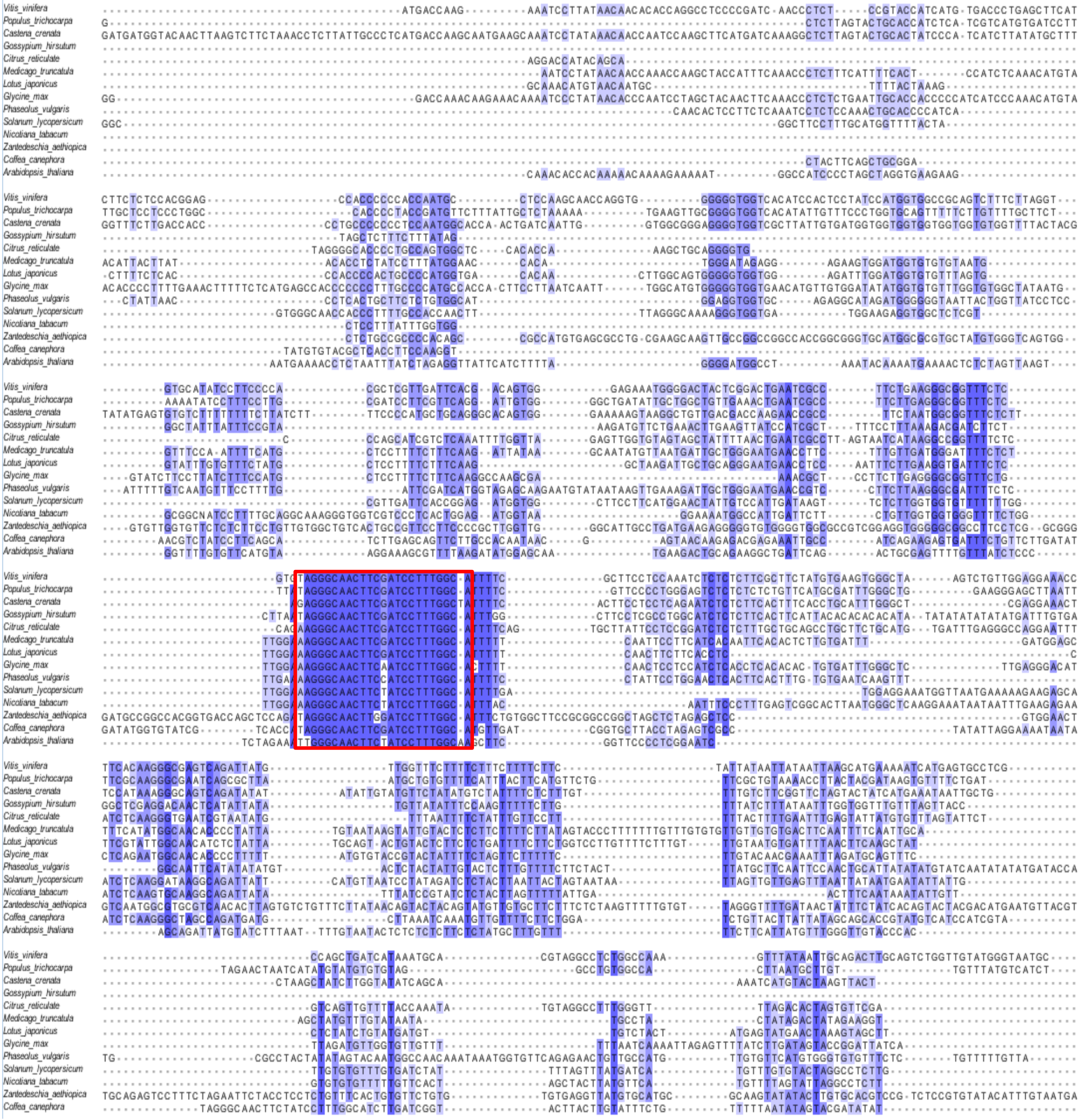
Vitis_vinifera
Populus_trichocarpa
Castena_crenata
Gossypium_hirsutum
Citrus_reticulate
Medicago_truncatula
Lotus_japonicus
Glycine_max
Phaseolus_vulgaris
Solanum_lycopersicum
Nicotiana_tabacum
Zantedeschia_aethiopica
Coffea_canephora
Arabidopsis_thaliana

Figure S2

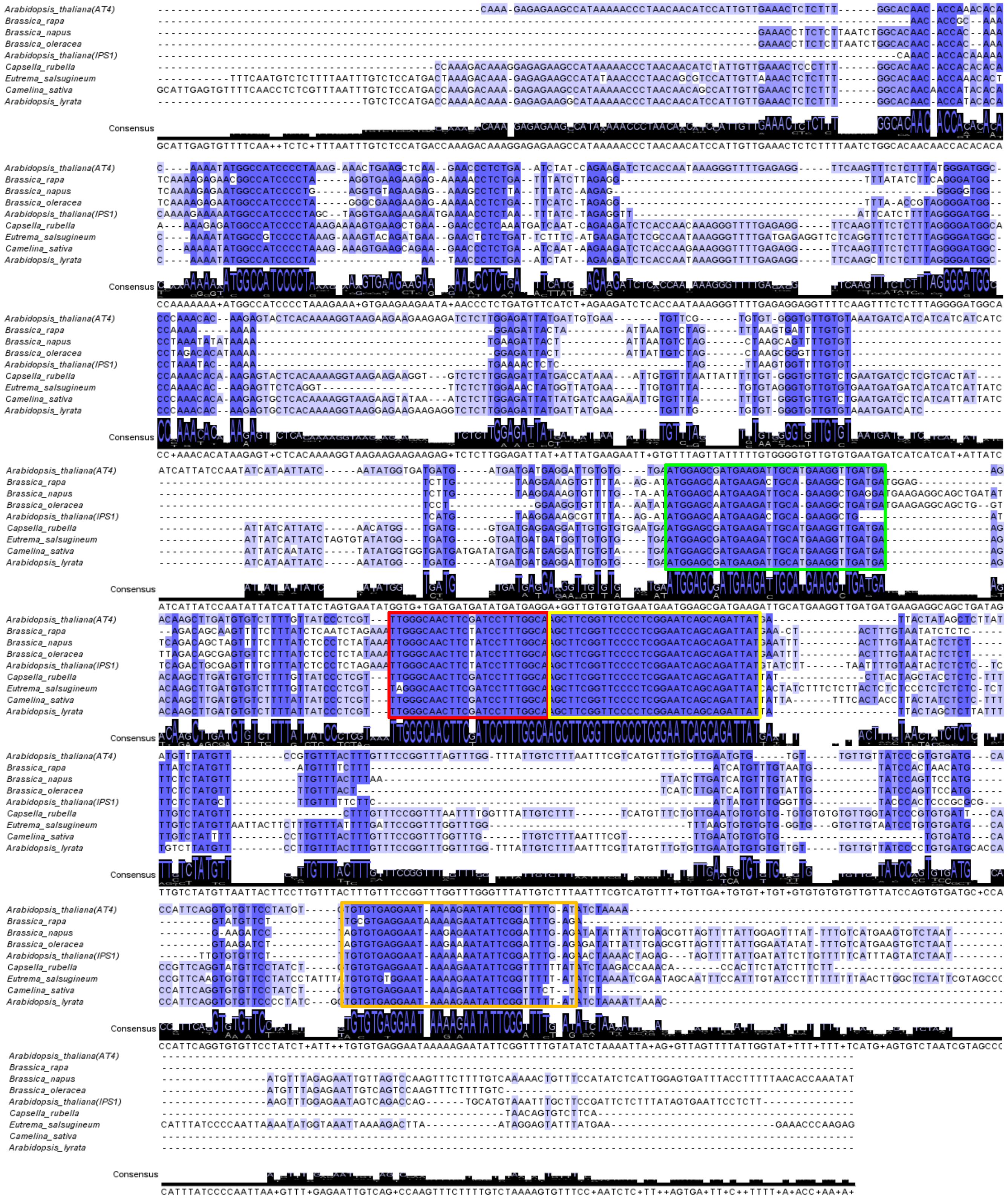
Arabidopsis_thaliana(AT4)
Brassica_rapa
Brassica_napus
Brassica_oleracea
Arabidopsis_thaliana(IPS1)
Capsella_rubella
Eutrema_salsugineum
Camelina_sativa
Arabidopsis_lyrata
Consensus

Figure S3

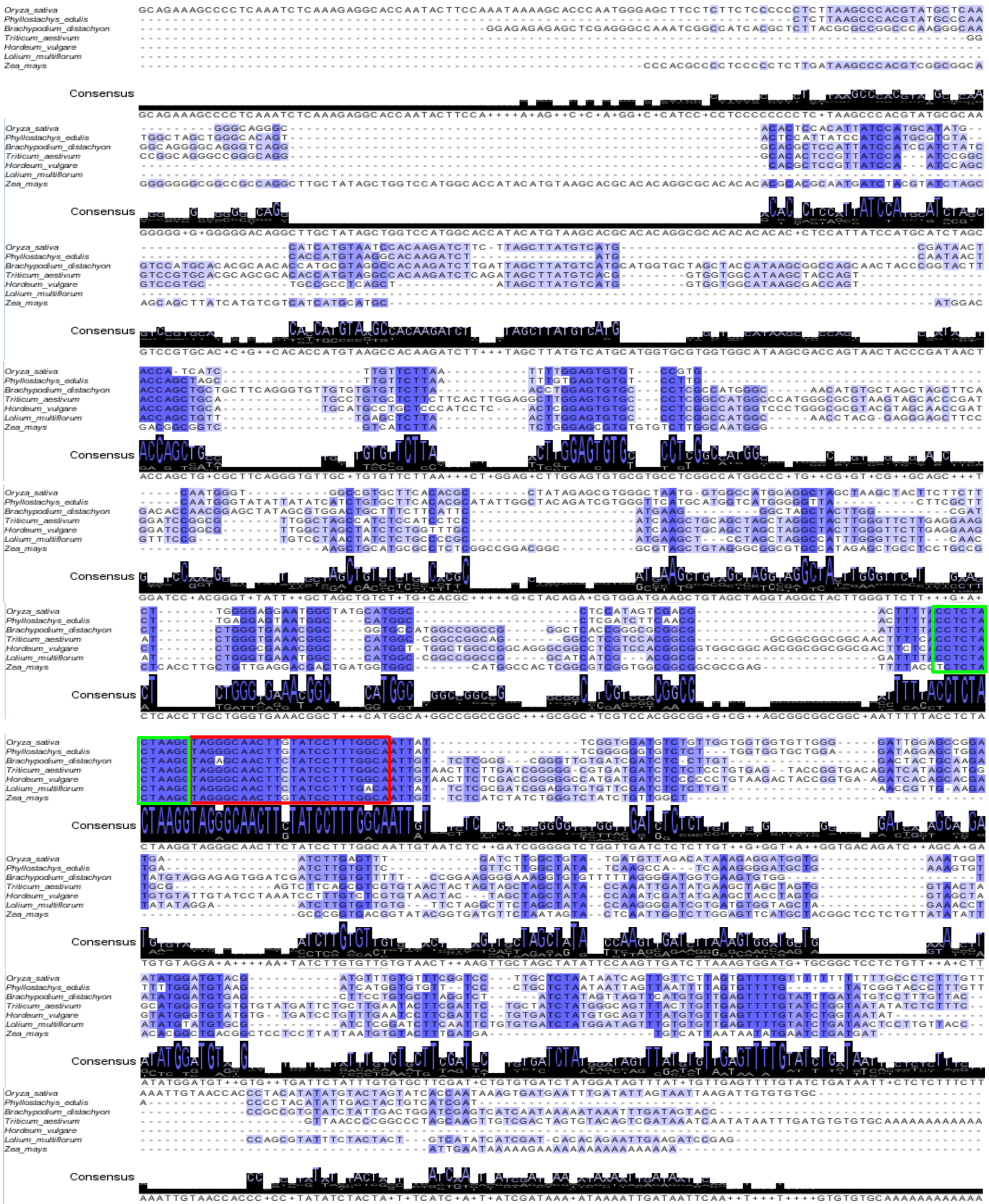

Oryza_sativa
Phyllostachys_edulis
Brachypodium_distachyon
Triticum_aestivum
Hordeum_vulgare
Lolium_multiflorum
Zea_mays
Consensus

Figure S4

```
Arabidopsis_thaliana   ----CAAACACCACAAAAACAAAAGAAAAATGGCCATCCCCTAGCTAGGTGAAGAAGA
Oryza_sativa           GCAGAAAGCCCCTCAAATCTCAAAGAGGCACCAATACTTCC---------------AA

Arabidopsis_thaliana   ATGAAAACCTCTAAT-------------------TTATCTAGAGGTTATTCATCTTTT
Oryza_sativa           ATAAAAGCACCCAATGGGAGCTTCCTCTTCTCCCCCCTCTTAAGCCCACGTATGCTCA

Arabidopsis_thaliana   AGGGGATGGC---------------------------CTAAATACAAAATGAAAACTC
Oryza_sativa           AGGGCAGGGCACACTCCACATTATCCATGCATATGCATCATGTAATCCACAAGATCTT

Arabidopsis_thaliana   TCTAGTTAA------------------GTGGTTTTGTGTTCATGTAAGGAAAGCGTTT
Oryza_sativa           CTTAGCTTATGTCATGCGATAACTACCATCATCTTGTTCTTAATTTTGGAGTGTGTCC

Arabidopsis_thaliana   -------------------------------TAAGATATGGAGCAATGAAGACTGCAG
Oryza_sativa           GTGCAATGGGTGGCCGTGCTTCACACGCCTATAGAGCGTGGCTAATGGTGGCCATGG

Arabidopsis_thaliana   A--------AGGCTGATTC-------------------------------------AG
Oryza_sativa           AGGCTAGCTAAGCTACTTCTTCTTCTTGGGGAGGAATGGCTATGCATGGCCTCCATAG

Arabidopsis_thaliana   ACTGCGAGTTTTGTTTATCTCCCTCTAGAAATTGGGCAACTTCTATCCTTTGGCAAGC
Oryza_sativa           TCGACGACTTTTA------CCTCTACTAAGGTAGGGCAACTTGTATCCTTTGGCAATT

Arabidopsis_thaliana   TT------------------CGGTTCCCCTCGGAATCAGCAGATTATGTATCTTTAAT
Oryza_sativa           ATTCGGTGGATGTCTGTTGGTGGTGGTGTTGGGGATTGGAGCCGGATGAATCTTGAGT

Arabidopsis_thaliana   TTTGTAATACTCTCTCTCTTCTCTATGCTTTGTTTTTCTTCATTATGTTTGGGTTGTA
Oryza_sativa           TTGAT---------------------------CTTGGCTGTATGATGTTAGA------

Arabidopsis_thaliana   CCCACTCCCGCGCGTTGTGTGTTCTTTGTGTGAGGAATAAAAAAATATTCGGATTTGA
Oryza_sativa           -----------------------CATAAAGAGGATGGTGAAATGGTATATGGATGTAC

Arabidopsis_thaliana   GA--------------------ACTAAAACTAGAGTAGTTTTATTGATATTCTTGTT
Oryza_sativa           GATGTTTGTGTTTCGGTCCTTGCTCTAATAATCAGTTGTTCTTAGTGTTTTGTTTTTT

Arabidopsis_thaliana   TTTCATTTAGTATCTAATAAGTT---------------TGGAGAATAGTCAGACCAG
Oryza_sativa           TTTTTTGCCCTCTTTGTTAAATTGTAACCACCCTACATATATGTACTAGTATCACCAA

Arabidopsis_thaliana   TGCA--TGTAAATTTGCTTCCGATTCTCTTTATAGTGAATTCCTCTT
Oryza_sativa           TAAAGTGATGAATTTGATATTAGTAATTAAGATTGTGTGTGC-----
```

Sequence identity = 49.93%

Figure S5

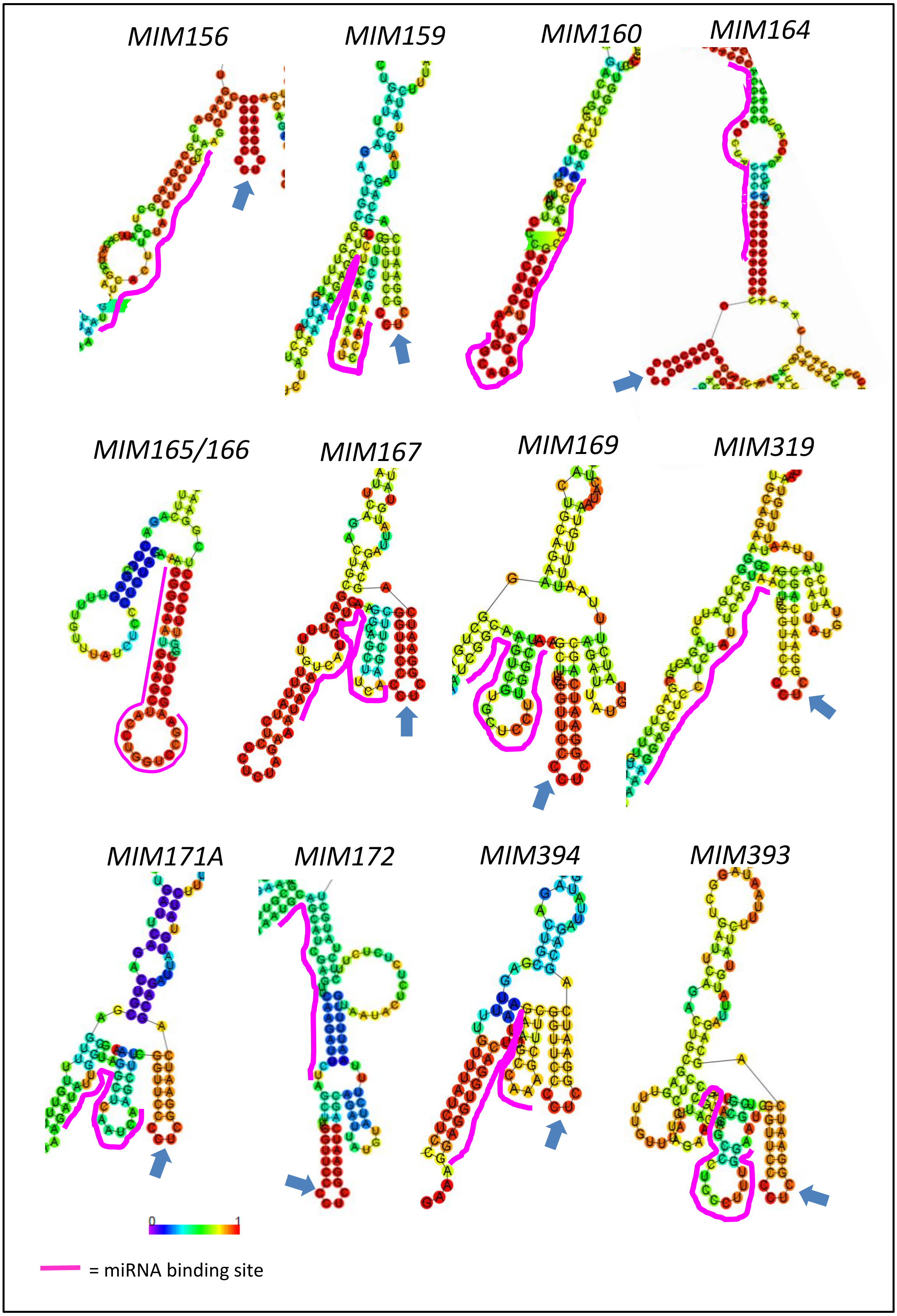
MIM156
MIM159
MIM160
MIM164
MIM165/166
MIM167
MIM169
MIM319
MIM171A
MIM172
MIM394
MIM393
0
1
= miRNA binding site

Figure S6

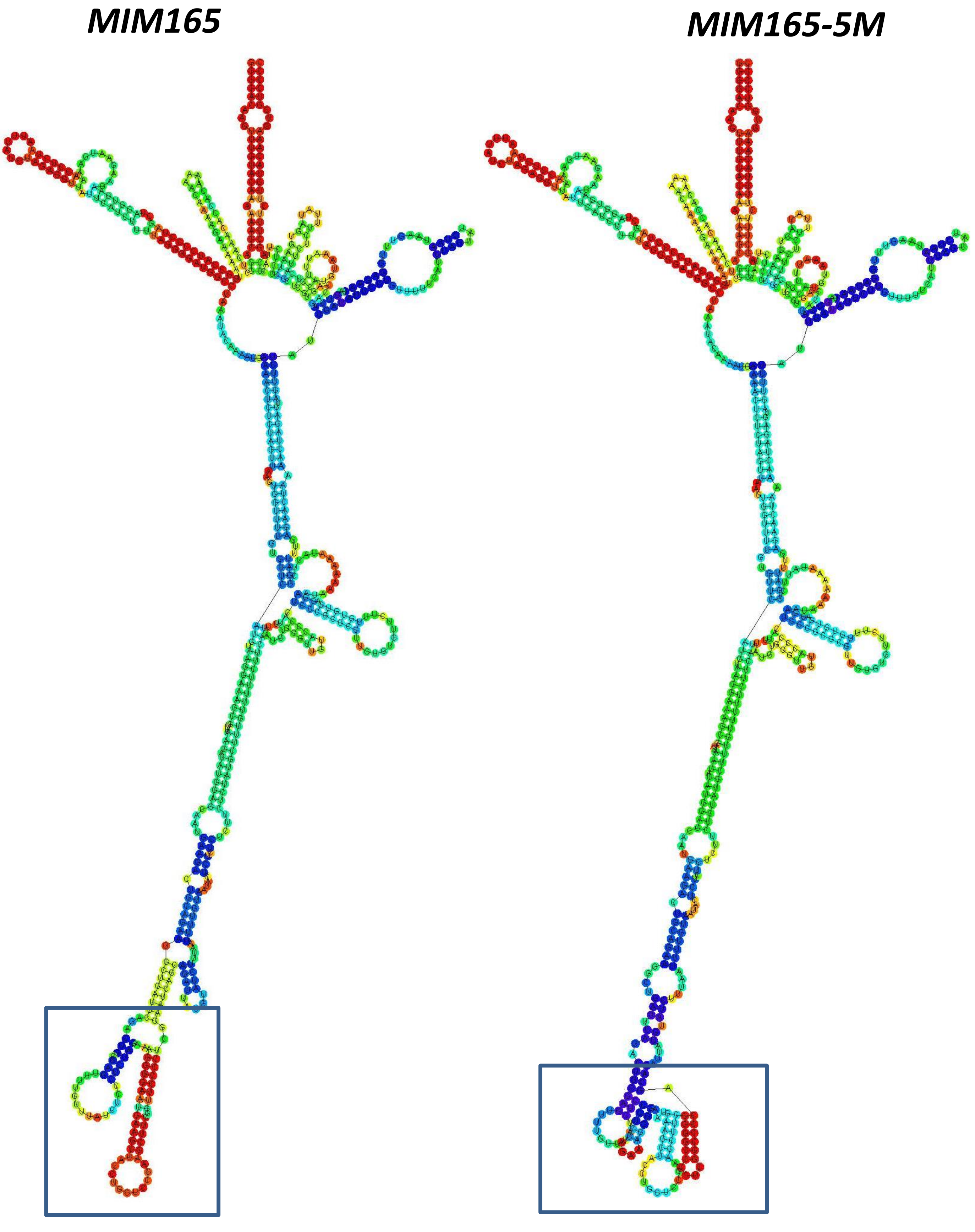

Supplement: Supplementary file 1 [file PLD3-2-e00088-s001.pdf]
