# Supplementary material for: MicroRNA MIMIC binding sites: Minor flanking nucleotide alterations can strongly impact MIMIC silencing efficacy in Arabidopsis
Source: Plant Direct. 2018 Oct 23;2(10):e00088. doi: 10.1002/pld3.88 (PMC6508833; doi:10.1002/pld3.88)
Supplement: Supplementary file 2 [file PLD3-2-e00088-s002.pdf]

Response to reviewers

Reviewer #1:

This research analyzed how the nucleoside(s) flanking the microRNA (miRNA)-binding site of target MIMICs (MIMs) affect on the inhibition of miRNA activity. MIMs is a tool to decoy miRNA through creating a mimic miRNA target. The authors found the presence of a conserved sequence existing downstream of miRNA-binding sites of IPS1, the founder RNA of MIMs, and showed that this sequence is important for MIMs function. Moreover, the authors showed the conserved sequence can potentially form a stem-loop structure, which is important for MIM165, but not for MIM159. These results show that the flanking sequences of miRNA-binding sites shall play critical roles in determining the activity of MIMs. The manuscript is well written.

No response is required here.

Reviewer #2:

In the current manuscript, Wong et al., study how MIMICRY flanking regions can influence their performance as miRNA decoys. The authors start their work as a follow up from two previous works from the same group, one showing the importance of regions flanking the miR159 target site within the MYB33 sequence, and one in which they compare the efficiency on miRNA sequestration from three different approaches (MIM, SP and STTM). In the latest, the authors concluded that the election of the most suitable method to downregulate the activity of a specific miRNA family is a one-by-one case. Several studies have shown that the MIM165 described in Todesco et al., (2010), is not as effective as STTM165 (Yan et al., 2012). Wong et al., describe the conservation of three additional regions within IPS1 homologs across the Brassicaceae family, two of them immediately flanking the miR399 decoy sequence. Nevertheless, when compared to IPS1 sequences from monocots, conservation beyond the miR399 decoy site is reduced to just 12pbs. To test the hypothesis that conserved flanking regions modulate decoy ability, the authors created versions of one of the most efficient MIMS, MIM159, in which they introduced different mutations within the conserved sequences flanking the MIM159 site. In addition, they built a MIM159 construct using as template IPS1 from rice (OsIPS1). Mutant versions as well as the one using OsIPS1, showed a reduced phenotypic penetrance when compared with MIM159. The authors assessed MIM, MYB 33 (a miR159 target) and CP1 (directly regulated by MYB33) transcript levels and found a correlation between phenotypic severity and MYB33 and CP1 expression. Surprisingly, they found that the levels of MIM159 transcripts where inverse correlated with the severity of the phenotype.

No response is required here.

Authors should check miR159 levels by either sRNA blot or mature miRNA qRT-PCR.

The reduced abundance of miR159 in MIM159 plants has been demonstrated in multiple previous publications (Reichel et al., 2015 Plant Biotech Journal; Reichel et al., 2015 J. Plant Physiol.). Although we acknowledge this would support the findings, we have already assayed MYB33 and CP1, which which indicate miR159 has been inhibited, similar to what has been previously demonstrated

(Alonso-Peral et al., 2010 Plant Physiol.; Li et al., 2014 PLoS Genetics; Li et al., 2016 BMC Plant Biol.). We have focused on the most important part of the experiment, that is the phenotyping of multiple MIM159 primary transformants, and we believe addition of this experiment will not alter the core findings/conclusions of the experiments.

In addition, authors should apply some statistic test to support which differences are significant (that applies to all data shown in Figs 2, 3 and 4).

Apologies for not including this in the original submission. This has now been performed and presented as requested by the reviewer and editor. For the phenotyping of primary transformants, the different constructs in Figures 2 and 3 were all significantly different from one another and these analyses have been added to the figures. For Figure 4, MIM159 and MIM159-MSL were found not to be significantly different. This does not alter any core finding or conclusion of the paper, but we have amended the text where appropriate to point this out. All appropriate statistics have been added to the qRT-PCR experiments. There are no error bars on Fig 3D, as there are only two biological replicates as indicated.

Later on, the authors studied whether there were secondary structures associated to the flanking conserved regions. All mRNAs have secondary structures that might be important for their translation/function. Using the RNAfold tool, the authors found a stem-loop structure adjacent to the MIM site for all the tested MIM constructs but MIM165. Therefore, the authors explored whether such difference could underlie the low efficacy of that MIM in sequestering miR165/6. To that end, the authors introduced mutations to restore the stem-loop next to the MIM165 site resulting in an increased efficacy on miR165 down-regulation, at least at the phenotypic level.

Nevertheless, the authors should assay miR165 levels by blot or qRT.

Again multiple previous publications e.g. (Yan et al., 2012 Plant Cell; Reichel et al., 2015 Plant Biotech. Journal) have demonstrated that expression of MIM165 represses miR165 miRNA levels, so we believe that performing this experiment will not alter the core findings/conclusions of the paper. Again the critical aspect is the phenotyping of multiple MIM165 primary transformants of the MIM165, MIM165-3m and MIM165-5m constructs.

Opposite to what they found when testing MIM159 expression levels, MIM65 levels where higher in backgrounds with stronger phenotype, questioning their conclusions from the MIM159 mutations in fig 2.

Actually the analysis of the MIM RNA levels was done differently between the two experiments.

For MIM159 (Figure 2), measurements were made in three pools of 14 randomly selected four-week old transformants to obtain the average RNA levels for each different MIM159 transgene (MIM159, MIM159-1m, MIM159-3m, OsMIM159). Between MIM159, MIM159-1m, MIM159-3m, it was found that the higher the efficacy the lower the average RNA level. This is pointed out and discussed in the paper.

By contrast, for MIM165, MIM165-3M and MIM165-5M, the analysis was done on RNA isolated from different phenotypic categories (none, moderate and severe) for each transgene. Although there was a positive correlation within each MIM165 variant, where the stronger the phenotype the higher the RNA level, comparison between different MIM165 variants, found the weaker variant (MIM165) had to be expressed at a much higher level than a stronger variant (e.g. MIM165-3M) to result in the same phenotypic outcome. This demonstrates that MIM165 has a weaker efficacy than MIM165-3M as pointed out in the manuscript.

Given these differences in how the MIM165 variants were analysed as compared to the MIM159 variants, the results of the MIM165 variants do not question the conclusions of the MIM159 variants.

Another difference with MIM159, is that when they tried to do the opposite and break the stem-loop structure next to the MIM159 sequence the mutations did not affect its decoy ability. Therefore, there are some inconsistencies between what the authors found in MIM159 and MIM165 analysis. There is no question that flanking regions might influence accessibility from a miRNA to its target sequence, but this referee is not sure that this can explain why MIM165 is not able to perform its role as efficient as other MIMs.

The reviewer is correct in that there are inconsistencies between MIM159 and MIM165 and the presence of the predicted stem-loop as being a determinant of efficacy. We have added the following to the discussion to sharpen our conclusion and to strengthen a possible explanation (which would require further experimentation) of the discrepancy of findings between MIM159 and MIM165;

"Given the conflicting results between *MIM165* and *MIM159*, the presence or absence of the predicted stem-loop cannot be regarded as an absolute indicator of efficacy. As mentioned above, the restoration of the stem-loop in *MIM165-3M* and *-5M* may have abolished a competing RNA secondary structure that was predicted to form in *MIM165* (Figure 3A), and which potentially inhibits the accessibility of the miRNA binding site. Therefore, in the *MIM165* context the stem-loop becomes an important determinant of silencing efficacy. By contrast, for *MIM159* no strong RNA secondary structure was predicted to sequester the miR159 binding site, even when the stem-loop is abolished (Figure 4A). Therefore, the stem-loop is not an important determinant of silencing efficacy in the *MIM159* context. Such a claim will need to be tested with further experimentation. Nevertheless, our observations highlight the complexity of miRNA binding sites, where changing only the miRNA binding site within the *MIM* backbone may not only change the miRNA that it targets, but potentially also the local RNA secondary structure that impacts miRNA target site accessibility, which ultimately impacts efficacy of the decoy."

One consideration might be that the target sequence in MIM165 is different to the one in STTM 165, with the possibility that the cleavage site is reconstituted due to the composition of the 3pbs bulge that interrupts the positions 10-11.

The MIM165 binding site used here is identical to the STTM165 binding site, including the composition of the 3 nt bulges (CTA), which was also used in the original paper describing MIM165 (Todesco et al., 2010). However, in this paper we are not comparing MIM165 to STTM165. The real comparison and difference is MIM165 to MIM165-3M and MIM165-5M. Therefore, as these features are identical, they cannot explain the difference in performance of the STTM165/166 compared to the MIM165. To clarify this, we have added the following text to the result section;

"The *MIM165* binding sites of the two variants remained unchanged from the binding site of the parental *MIM165* transgene, and all three binding sites were identical to the STTM165 binding site (Yan et al., 2012), including the three nucleotide bulge (CTA) at position 10-11 (Todesco et al., 2010)."

Is there a stem-loop in the STTMs and SPs that can also explain their differential ability to sequester miRNAs?,

For the STTM design, yes there is this potential, and we noted this in the discussion of the original submission (page 12, line 273); "Such a principle was hypothesised to be behind the strong efficacy of *STTM165/166* (Yan et al., 2012), where this decoy is composed of a 48 nt spacer region, that potentially forms a strong RNA secondary structure, that is flanked by two *MIM165* binding sites."

However, for the cmSP165/166 that has strong efficacy, the 15 binding sites are only separated by 4 nt spacers. It could be that strong secondary structures are forming in this decoy, and only some binding sites are sequestering the miRNA, but such a claim is pure speculation so we do not think adding a sentence to point this out will add anything of substance to the discussion.

If not, what is really the importance of such structure when avoiding its formation in the engineered MIM159 is not affecting its performance?

I think this question is now answered in the discussion that we have added to address a previous above question, i.e.

*"*Given the conflicting results between *MIM165* and *MIM159*, the presence or absence of the predicted stem-loop cannot be regarded as an absolute indicator of efficacy. As mentioned above, the restoration of the stem-loop in *MIM165-3M* and *-5M* may have abolished a competing RNA secondary structure that was predicted to form in *MIM165* (Figure 3A), and which potentially inhibits the accessibility of the miRNA binding site. Therefore, in the *MIM165* context the stem-loop becomes an important determinant of silencing efficacy. By contrast, for *MIM159* no strong RNA secondary structure was predicted to sequester the miR159 binding site, even when the stem-loop is abolished (Figure 4A). Therefore, the stem-loop is not an important determinant of silencing efficacy in the *MIM159* context. Such a claim will need to be tested with further experimentation. Nevertheless, our observations highlight the complexity of miRNA binding sites, where changing only the miRNA binding site within the *MIM* backbone may not only change the miRNA that it targets, but potentially also the local RNA secondary structure that impacts miRNA target site accessibility, which ultimately impacts efficacy of the decoy."

Is the stem-loop consistently present when the entire IPS1 backbone is taking into account for RNA secondary structure modelling?,

Yes, the structures are consistent when the entire IPS1 backbone is include. We have now included the entire structures in Supplementary data (Figure S6) and indicated this in a sentence in the text; "The structures were present when the entire *MIM165* RNAs were folded (Figure S6)."

How many possible structures is RNAfold retrieving in any case (the more structures retrieves, the less stable those structures are).

We had only used the Vienna RNAfold program which only presents the most probable structure. We have now also used the mFold program which lists the number of possible structures. The complexity

here is even if a RNA has multiple structures, this number of structures does not reflect the stability of the stem-loop region. For example, mFold predicted 17 different structures for MIM165-5M, but all 17 had the predicted stem-loop. In fact, the frequency of the stem-loop being predicted is tightly correlated with the confidence at which the Vienna RNA fold program predicted the stem-loop (see Table below). Given that both programs use the minimum free energy (MFE) algorithm based on Zuker, this consistency is not surprising. We were considering adding the Table to the manuscript, but thought that this is really not adding anything to the manuscript, so decided against it. If you feel otherwise we are happy to add it.

**Gene**	**No. of structures with stem-loop predicted**	**No. of secondary structure retrieved**	**Percentage of structures with predicted stem-loop (%)**
AtIPS1	15	18	83
MIM165/166	5	22	22
MIM165-3M	16	18	88
MIM165-5M	17	17	100
MIM159	11	14	78
MIM159-1m	0	13	0
MIM159-3m	0	23	0
MIM159-MSL	0	16	0
MIM159-RES	12	15	80

Minor comments:
- Page 5, line 96; replace splicing by slicing
- Page 8, line 167; replace "a least" by "at least"
- Page 16, line 343; replace Todseco by Todesco

All minor edits done.
